# Supplementary material for: Empirical methods for controlling false positives and estimating confidence in ChIP-Seq peaks
Source: BMC Bioinformatics. 2008 Dec 5;9:523. doi: 10.1186/1471-2105-9-523 (PMC2628906; doi:10.1186/1471-2105-9-523)
Supplement: Additional File 1 — A variety of html documents from the USeq web site detailing the available applications, their best usage, output file type descriptions, command line menus, etc. [file 1471-2105-9-523-S1.zip › USeqUserGuides/JavaDoc.zip!/JavaDoc/trans/bkgrnd/MapControlIntensities.html]

MapControlIntensities


|  |  |  |
| --- | --- | --- |
| **Overview** \| **Package** \| **Class** \| **Use** \| **Tree** \| **Deprecated** \| **Index** \| **Help** | |  |
| **PREV CLASS**   NEXT CLASS | **FRAMES**    **NO FRAMES**     **All Classes** |  |
| SUMMARY: NESTED \| FIELD \| CONSTR \| METHOD | DETAIL: FIELD \| CONSTR \| METHOD |  |


---


## trans.bkgrnd Class MapControlIntensities

```
java.lang.Object
  trans.bkgrnd.MapControlIntensities
```

---

``` public class MapControlIntensities extends java.lang.Object ```

Loads a bpmap file with oligo intensity measurements from multiple '.cel' files after median scaling to 50.

---

| **Field Summary** | |
| --- | --- |
| `static float` | `TARGET_MEDIAN` |


**Constructor Summary**

`MapControlIntensities()`


| **Method Summary** | |
| --- | --- |
| `static void` | `loadIntensities(BackGroundOligo[] oligos, java.io.File[] celFiles)` |
| `static void` | `main(java.lang.String[] args)` |
| `static BackGroundOligo[]` | `makeInitialBGOs(java.io.File bpmap)` |

**Methods inherited from class java.lang.Object**

`equals, getClass, hashCode, notify, notifyAll, toString, wait, wait, wait`

**Field Detail**

### TARGET\_MEDIAN

```
public static final float TARGET_MEDIAN
```

**See Also:**: Constant Field Values


**Constructor Detail**

### MapControlIntensities

```
public MapControlIntensities()
```


**Method Detail**

### main

```
public static void main(java.lang.String[] args)
```

---


### loadIntensities

```
public static void loadIntensities(BackGroundOligo[] oligos,
                                   java.io.File[] celFiles)
```

---


### makeInitialBGOs

```
public static BackGroundOligo[] makeInitialBGOs(java.io.File bpmap)
```


---


|  |  |  |
| --- | --- | --- |
| **Overview** \| **Package** \| **Class** \| **Use** \| **Tree** \| **Deprecated** \| **Index** \| **Help** | |  |
| **PREV CLASS**   NEXT CLASS | **FRAMES**    **NO FRAMES**     **All Classes** |  |
| SUMMARY: NESTED \| FIELD \| CONSTR \| METHOD | DETAIL: FIELD \| CONSTR \| METHOD |  |


---
